# The growth threshold conjecture: a theoretical framework for understanding T-cell tolerance

**DOI:** 10.1098/rsos.150016

**Published:** 2015-07-08

**Authors:** Clemente F. Arias, Miguel A. Herrero, José A. Cuesta, Francisco J. Acosta, Cristina Fernández-Arias

**Affiliations:** 1 Departamento de Matemática Aplicada, and, Universidad Complutense de Madrid, Madrid, Spain; 2 Departamento de Ecología, Universidad Complutense de Madrid, Madrid, Spain; 3 Grupo Interdisciplinar de Sistemas Complejos, Madrid, Spain; 4 Departamento de Matemáticas, Universidad Carlos III de Madrid, Leganés, Madrid, Spain; 5 Instituto de Biocomputación y Física de Sistemas Complejos (BIFI), Universidad de Zaragoza, Zaragoza, Spain; 6 Department of Microbiology, Division of Parasitology, New York University School of Medicine, New York, NY, USA

**Keywords:** T cells, immune tolerance, negative selection, immunodominance, immune self

## Abstract

Adaptive immune responses depend on the capacity of T cells to target specific antigens. As similar antigens can be expressed by pathogens and host cells, the question naturally arises of how can T cells discriminate friends from foes. In this work, we suggest that T cells tolerate cells whose proliferation rates remain below a permitted threshold. Our proposal relies on well-established facts about T-cell dynamics during acute infections: T-cell populations are elastic (they expand and contract) and they display inertia (contraction is delayed relative to antigen removal). By modelling inertia and elasticity, we show that tolerance to slow-growing populations can emerge as a population-scale feature of T cells. This result suggests a theoretical framework to understand immune tolerance that goes beyond the self versus non-self dichotomy. It also accounts for currently unexplained observations, such as the paradoxical tolerance to slow-growing pathogens or the presence of self-reactive T cells in the organism.

## 1. Introduction

The ability of the adaptive immune system to target specific cells is mediated by an elaborate mechanism of antigen recognition. T cells interact with antigens through a membrane receptor (the T-cell receptor or TCR) that binds to small antigenic regions called epitopes. The TCR has a clonal distribution, i.e. different individual T cells may carry multiple copies of just one type of TCR. Nevertheless, the precise structure of the TCR and hence the set of antigens it can bind varies between clones. At any given time, a great diversity of clones coexist in the organism. This allows for the potential recognition of a huge range of antigens and therefore ensures that virtually any pathogen can be the target of a T-cell immune response. Importantly, T cells not only respond to external pathogens. In fact, antigens are also present in host cells, so that T cells can target self-antigens in normal physiological processes [1] and detect and kill tumour cells growing within host tissues [2]. The affinity of T cells for self-antigens can also lead to autoimmune diseases in some cases. On the other hand, non-self antigens such as those arising from non-pathogenic commensal and symbiotic bacteria in the gut are often tolerated by T cells [3]. In fact, some antigens may be tolerated in the organism or become instead the target of a T-cell response depending on the circumstances. This happens, for instance, when host and pathogen antigens share epitopes and can therefore be recognized by the same TCRs. Clones with affinity for such epitopes may cause autoimmunity in the presence of a pathogen by cross-reacting against otherwise tolerated host cells [4]. These remarks show that T-cell response against an antigen does not depend on its molecular structure or genetic origin, which naturally raises the question of how T cells discriminate pathogenic antigens and tolerate non-pathogenic ones.

It has been suggested that variations in the quantities of a given antigen (either self or non-self) in the organism might trigger the activation of T cells [5]. In agreement with this approach, we show in this work that upon activation, T-cell population dynamics provides a mechanism that allows for the immune discrimination of antigens based on their rates of variation. In particular, we postulate that T cells tolerate cognate antigens (whether pathogenic or not) whose rate of production remains below a given threshold. From this perspective, immune tolerance is not an intrinsic property of antigens, but is determined by the population dynamics of cells where antigens originate. In fact, antigens with high production rates are expected to be associated with pathogenic toxins or structural proteins of either infectious agents or aggressive tumour cells, characterized by their high proliferative ability. According to this view, cells carrying such antigens (both host or external) will be targeted as foes by T cells. Our proposal is motivated by two features of the T-cell population dynamics during immune response that are conspicuous in acute infections: inertia and elasticity.

## 2. Inertia and elasticity in T cells

T cells circulate through the blood and lymph systems in a resting or naive state. In case of an infection, innate immune cells loaded with pathogen antigens collected from the infected tissues interact with naive T cells in the lymph nodes. Naive T cells whose TCR recognizes some of those antigens activate, differentiate into effector T cells and undergo clonal expansion, a rapid proliferation promoted by antigenic stimulation that usually results in elimination of the pathogen. Once this has occurred, effector T cells eventually disappear in a process termed clonal contraction that restores initial population levels [6]. Only a few cells survive as memory T cells after clonal contraction, reverting to a quiescent state and providing the potentiality of a rapid response in the event of a re-infection by the same agent [7,8].

**Figure 1.**
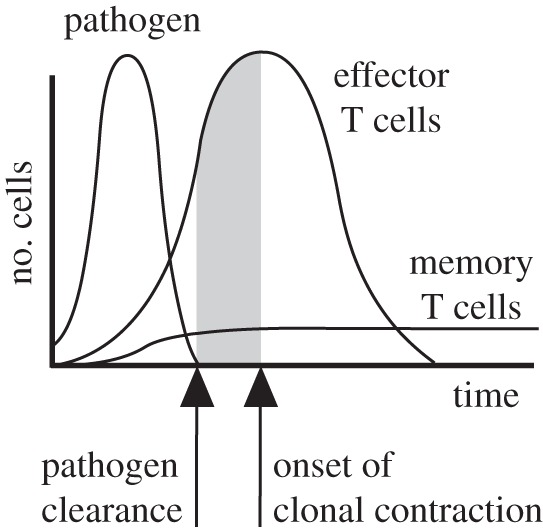
Standard description of T cells and pathogen dynamics during an acute infection. Clonal expansion induced by the pathogen in the population of effector T cells continues once the infection has been controlled, resulting in a delayed onset of clonal contraction.

Clonal expansion and contraction can be understood as emerging, population-level features resulting from individual T cells choices to divide or die by apoptosis after activation [9]. Actually, the complexity of the cellular events underlying the timing of clonal contraction and expansion is in sharp contrast with the deceiving simplicity of their macroscopic description. Roughly speaking, the population of effector T cells can be thought of as expanding in response to an antigenic stimulation, and as reverting to its initial state (owing to a massive cell death and/or to a transition to a memory phenotype) when the antigen is no longer present. However, it distinctly shows inertia, as expansion continues in the absence of antigen, thus leading to a delay in the onset of clonal contraction relative to the disappearance of the pathogen [10] (figure 1). Based on these remarks, we will next show that such a macroscopic description admits a simple mathematical formulation that captures essential features of T-cell response and allows us to account for behaviours that remain unexplained so far.

To begin with, our model in equations (2.1) makes use of second-order ordinary differential equations (ODEs). These provide what is arguably the simplest mathematical setting that yields inertial effects on a population (of effector T cells in our case). Moreover, second-order ODEs are the natural framework for Newtonian dynamics. This naturally leads us to model effector T-cell dynamics as driven by the balance between two opposite forces acting on the population: an antigenic force caused by the presence of the pathogen and an intrinsic elastic force that tends to bring the population back to its initial state. Specifically, we will assume the antigenic force to be proportional to the number of pathogens and will model the elastic, recovery force by means of Hooke's Law in mechanics [11]. The latter states that the force required to restore equilibrium once a population has reached a given value is proportional to such value. We will also assume that pathogens proliferate at a constant rate and are eliminated by effector T cells proportionally to their mutual encounters. These assumptions lead to the following model:

2.1 $$T^{\prime \prime }(t)=-kT(t)+\lambda P(t)\;\mathrm{and}\;P^{\prime }(t)=\alpha P(t)-\beta T(t)P(t),$$

where *T*(*t*) and *P*(*t*) are, respectively, the number of effector T cells and pathogens at time *t* and *k*, λ, *α* and *β* are positive parameters. Equations (2.1) model the dynamics of the effector T-cell population from the instant of naive T-cell activation until the end of clonal contraction (see the electronic supplementary material, §A. The code for numerical simulations in Wolfram Mathematica software is provided in the electronic supplementary material, §C). We assume that the infection is controlled when the pathogen population falls below a given threshold that represents the minimum population size for which the pathogen is still infective.

**Figure 2.**
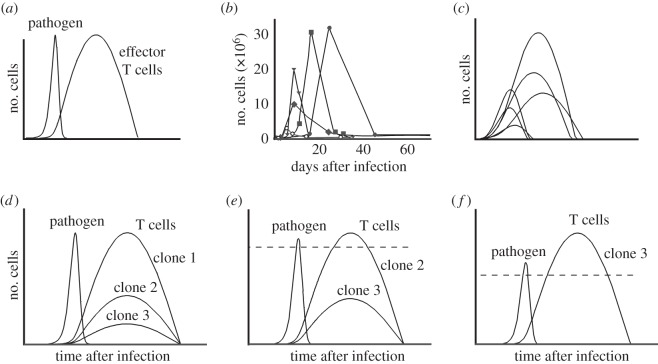
Numerical simulations of the model. (*a*) Equations (2.1) capture the qualitative dynamics of effector T cells and pathogen populations. (*b*) Experimental data available in the literature [12–14] show differences in the peak of clonal expansion and in the duration of the T-cell immune response. (*c*) The observed variability in T-cell dynamics can be modelled by changing the value of parameters in equations (2.1). (*d*) The model shows that clones with different affinities for antigens may vary in the magnitude of clonal expansion during their simultaneous response to a pathogen. (*e*,*f*) Simulating the absence of dominant clones results in the control of the infection owing to compensatory expansion of subdominant clones. The dashed line marks the peak of clonal expansion reached by clone 1 in (*d*).

Numerical simulations of equations (2.1) reveal a number of interesting features. First, they reproduce clonal expansion and contraction (figure 2*a*), as well as the delay in the onset of clonal contraction and the observed variability in both the peak of clonal expansion and the duration of the T-cell response [12–14] (figure 2*b*,*c*). Furthermore, model (2.1) can be readily extended to consider the simultaneous response of several clones of T cells owing to the presence of different epitopes in a pathogen [15]. To do that, we just assume that clones interact with the pathogen in proportion to their respective abundances (see the electronic supplementary material, §B, for details). As in the case of equations (2.1), numerical simulations reveal that the resulting model displays clonal expansion and contraction of activated clones. Moreover, it also accounts for the empirical observation that clones with higher affinities for pathogen antigens (called immunodominant) expand significantly more than others during an immune response [15,16] (figure 2*d*). In addition, the model predicts that if immunodominant clones are removed, subdominant ones may expand to replace them and thus control the infection (figure 2*d*,*f*). It is worth noticing that recent empirical evidence suggests the existence of such compensatory expansion in T cells [17]. Altogether, the previous facts show that the proposed framework succeeds in reproducing key qualitative features of effector T-cell response. We will show next that it also suggests a potential mechanism allowing T cells to discriminate target cells according to their growth rate.

## 3. The growth rate threshold conjecture

As we have seen above, the existence of inertia in the T-cell immune response is supported by the delay in the onset of clonal contraction during acute infections. However, in some cases, clonal contraction can also take place before the disappearance of cells carrying the antigen [10,18]. This allows for target cells to escape the immune response, which in turn entails the subsequent reactivation of memory T cells of the same clones. For reasons that remain currently unexplained, the recurrence of this process may result in a progressive exhaustion of these clones, thus leading to chronic infections or tumours [8,19]. We remark that early contraction, together with the exhaustion of activated clones result in the eventual tolerance of the cell population that triggered the T-cell response.

**Figure 3.**
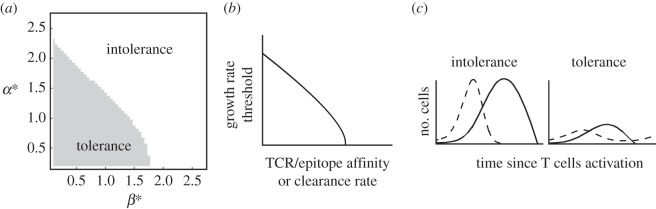
Parameter dependence of tolerance in equations (2.1). (*a*) Depending on the values of the parameters, the outcome of equations (2.1) can be either the elimination of the target population or its tolerance by early contraction. Parameters α* and β* correspond to the non-dimensional form of equations (2.1) (see the electronic supplementary material for details). (*b*) This result allows us to define a growth rate threshold that determines tolerance by early contraction as a function of clone features such as the affinity of its TCR for its cognate epitope or its target clearance rate. According to the model, higher affinities imply less tolerance to target cell populations. (*c*) Simulations of the immune response of a clone of effector T cells against two target populations with different growth rates show that clearance of the fast-growing population is accompanied by a greater clonal expansion. This suggests that tolerance by early contraction is not caused by a limit in the capacity of effector T cells to expand as has been suggested in the literature [10].

In our model, early contraction can be retrieved from analysis of equations (2.1). Indeed, a detailed exploration of the behaviour of equations (2.1) delimits a region of parameters that lead to tolerance by early contraction (see the electronic supplementary material, §C, and figure 3*a*). This result can be given the following biological interpretation. Upon activation, effector T cells locate and kill cells displaying their cognate antigens. High TCR/antigen affinities or high clearance rates result in a potent T-cell immune response that eradicates target cells independently of their growth rate. However, for clones with lower affinities or reduced clearance rates, a growth rate threshold exists that determines if antigen-carrying cells remain after clonal contraction (figure 3*b*). In this case, the same clone can produce a strong immune response against fast-growing populations and tolerate populations with low growth rates. According to equations (2.1), the persistence of a slow-growing population is not owing to a limit in the expansive capacity of effector T cells, as has been suggested in the literature [10]. This is shown by the greater expansion exhibited by the same clone in an acute response (figure 3*c*). Instead, early contraction can be explained in terms of the framework introduced above as occurring because the antigenic force exerted by a slow-growing population cannot outbalance the elastic force of effector T cells, and thus fails to maintain a sustained clonal expansion. This interpretation is compatible with the observed premature death of clones targeting viral antigens in hepatitis C virus and norovirus chronic infections [19,20].

## 4. T-cell response revisited

In this section, we will show that the growth threshold conjecture provides a coherent view of current knowledge about T-cell biology. The repertoire of circulating naive T cells is known to emerge as the result of two opposite processes called positive and negative selection. T-cell precursors interact with a variety of peptides in the thymus and, depending on the affinity of their TCRs for these peptides, they survive and differentiate into functional naive T cells or die by apoptosis. Negative (respectively, positive) selection is defined as the deletion of precursor cells with high (respectively, low) affinity for some of these peptides [21,22]. Thus, only precursor T cells with an intermediate affinity for self-antigens are recruited to join the pool of naive T cells [23].

In our view, positive selection might be related to immunodominance: as clones with low affinities will not expand significantly in the presence of dominant clones (figure 2*d*), their deletion during positive selection guarantees that only sufficiently reactive clones will activate during an immune response. On the other hand, negative selection is currently considered as a mechanism that prevents autoimmunity by ensuring that highly self-reactive T cells do not circulate in the organism [21,24,25]. This shows that tolerance is implicitly assumed to depend on the low affinity of TCRs for self-antigens. This interpretation is not completely satisfactory though, given that self-reactive T cells with low affinities for their cognate epitopes can still target host cells and lead to autoimmunity [4,26]. Furthermore, compensatory proliferation of subdominant clones shows that T cells with low antigenic affinity can also generate effective immune responses against external pathogens [17].

**Figure 4.**
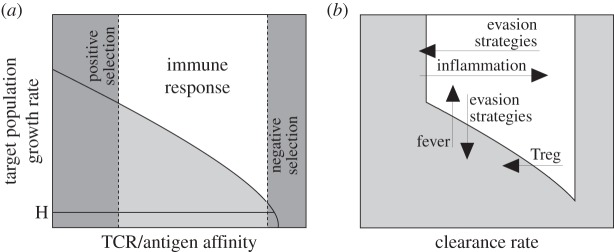
The growth threshold conjecture as a unifying framework. (*a*) Positive and negative selection can tune the growth rates that will be tolerated by T cells. By deleting clones with high affinity for (self or non-self) antigens, negative selection ensures that T cells tolerate target populations with low growth rates. Tolerance to host cells requires the survival of only those clones responding to growth rates greater than the upper bound of host-tissues turnover rates in normal homeostasis (H). (*b*) T-cell tolerance is determined by the population dynamics of effector T cells and target cells after activation. For a given clone within the range of TCR/epitope affinities defined by positive and negative selection, tolerance by early contraction (grey colour in the figure) or intolerance (white colour) are determined by target population growth and clearance rates. Mechanisms affecting these parameters can further modulate T-cell tolerance. For instance, pathogens and tumours can escape the action of T cells by reducing clearance rates (e.g. by immunosuppression) or by reducing their proliferation rates. Inflammation and fever increase clearance rates and proliferation rates, respectively, thus inducing intolerance. Regulatory T cells (Treg) foster tolerance by competing for antigen with effector T cells.

The growth threshold conjecture suggests an alternative explanation for the role of negative selection: its goal is not to discriminate clones according to their affinity for self versus non-self antigens. Instead, its serves to calibrate the growth rate thresholds that will be tolerated by effector T cells (figure 4*a*). Figure 3*a*,*b* shows that T cells with high affinity for their cognate antigen have no tolerance for cells carrying those antigens. Based on this result, we postulate that such clones are not deleted because they may target self antigens, but to provide effector T-cell tolerance to a range of proliferation rates. We remark that, in order for tolerance to host cells to be compatible with the existence of self-reactive clones, it suffices for growth thresholds determined by negative selection to be greater than the turnover rates of host cells in normal homeostasis (figure 4*a*). From this perspective, self-reactive clones may activate during an immune response to pathogens owing, for instance, to the presence of self antigens from necrotic host tissues in innate cells arriving at the lymph nodes from the site of the infection [27,28]. However, according to the growth threshold conjecture, these clones will undergo early contraction and therefore tolerate host cell populations.

According to equations (2.1), mechanisms affecting the clearance rate of the target of an immune response can further modulate tolerance to both host or external cells (figure 4*b*). For instance, inflammation increases the efficiency of the T-cell response and can lead to loss of self-tolerance in case of tissue injury or homeostatic imbalance [29,30]. By contrast, a specific set of T cells, called regulatory T cells, can induce tolerance by competing with reactive clones for access to antigenic stimulation, thus decreasing the clearance rate of target cells [31,32]. Similarly, pathogens and tumour cells can reduce the clearance rate by manipulating host cells death machinery and escape the action of T cells [33].

Interestingly, equations (2.1) suggest that tolerance can also be regulated by changes in the rate of growth of potential target populations (figure 4*b*). In fact, slow growth has been described in the literature as a paradoxical immune evasion strategy adopted by dormant tumour cells [34] and by many pathogens that seem to be associated with chronic infections [35,36]. Equations (2.1) also suggest a potential role of fever during infections. Fever is currently considered as enhancing the performance of host defences [37]. From the perspective of the growth threshold conjecture, fever might also serve to increase the proliferation rate of pathogen populations, thus increasing the efficiency of T-cell immune response [38].

## 5. Discussion

The growth threshold conjecture points to the dynamics of T cells and antigen-carrying cells as relevant factors of immune tolerance. According to this conjecture, effector T cells may consider as ‘self’ those cell populations growing at physiological rates, even if they are pathogenic. On the other hand, host cells can be perceived as ‘non-self’ and become the target of a T-cell autoimmune response in case of an abnormal increase in their proliferation rates [29]. The importance of qualitative or quantitative variations in antigen availability on immune tolerance has been discussed in previous works (see, for instance, [5,39,40]). However, the mechanism underlying the ‘immune perception’ [41] of antigenic variation introduced here differs form those described in the literature in two important aspects.

First, the discrimination of antigenic growth rates relies on effector T cells, i.e. it takes place after T-cell activation. By contrast, available models of T-cell tolerance refer to previous stages of the immune response. For instance, the discontinuity theory of immunity [5] aims to explain the activation of innate immune cells as owing to sharp changes in their environment. These cells are equipped with membrane-bound receptors, generically known as pattern recognition receptors, that recognize a wide variety of molecular patterns usually expressed by pathogenic agents, as well as ‘danger’ signals released by host cells in case of cellular stress [42,43]. According to the discontinuity theory, a sudden modification in the quantity of these signals can trigger the initiation of an immune response. Conversely, long-lasting modifications may result in the adaptation of innate cells, thus leading to tolerance. Alternative models, such as the tunable activation threshold model [44,45] or the quantal theory of immunity [46], link T-cell tolerance to the decisions of individual naive T cells of whether to activate or not upon their interactions with antigen-presenting cells. The rate at which antigen is presented to a naive T cell, together with the integration of excitation and de-excitation signals from the cell context may determine the responsiveness of the T cell and, consequently, the balance between tolerance or response to particular antigens. We remark that the growth threshold conjecture does not oppose any of these models. In fact, considering the complexity of the immune response, tolerance to host tissues is likely to be simultaneously controlled by different elements of the immune system. In this context, the growth threshold conjecture provides an explicit mechanism by which autoimmunity against normal, healthy host tissues can be avoided, even if other mechanisms of tolerance fail and self-reactive T cells activate.

A second difference between our model and other theories of immune tolerance is that the perception of antigenic growth rates emerges as a population-level feature. While the decision to activate can be taken by individual naive T cells based on the information provided by local clues and antigen-presenting cells [39,46], the early onset of clonal contraction leading to tolerance cannot be decided by any single effector T cell. Each effector T cell has very limited information about the progression of an immune response and it is unlikely that any individual effector T cell can measure the growth rate of cells carrying its cognate antigen. However, collective features such as inertia and elasticity might allow for the population of effector T cells as a whole to discriminate growth rates and choose between an acute response or tolerance.

The growth threshold conjecture also provides a sound explanation for the role of T-cell negative selection. Immune tolerance by early contraction does not require the recognition of self antigens by individual T cells. From this view, antigens are not used as marks of self or non-self, but to discriminate the growth rates of cells carrying specific antigens. Accordingly, negative selection does not need to be explained as a process that shapes the immune self as a collection of host peptides. Instead, it might serve to set the proliferation rates that will be tolerated by the immune system.

## Supplementary Material

elasticity OPEN SCIENCE supp mat.pdf

## Data accessibility

The code for numerical simulations of equations (2.1) in Wolfram Mathematica 7 is available in the last section of the electronic supplementary material of this article.
