# Supplementary material for: The growth threshold conjecture: a theoretical framework for understanding T-cell tolerance
Source: R Soc Open Sci. 2015 Jul 8;2(7):150016. doi: 10.1098/rsos.150016 (PMC4632576; doi:10.1098/rsos.150016)
Supplement: elasticity OPEN SCIENCE supp mat.pdf [file rsos150016supp1.pdf]

Supplementary Information for: The growth threshold conjecture: a theoretical framework for understanding T cell tolerance

Clemente F. Arias[1,2*], Miguel A. Herrero[1], José A. Cuesta[2,3,4], Francisco J. Acosta [5] and Cristina Fernández-Arias[6]

[1]Departamento de Matemática Aplicada. Universidad Complutense de Madrid. Madrid, Spain
[2] Grupo Interdisciplinario de Sistemas Complejos
[3]Departamento de Matemáticas. Universidad Carlos III de Madrid. Leganés, Madrid, Spain
[4]Instituto de Biocomputación y Física de Sistemas Complejos (BIFI). Universidad de Zaragoza, Zaragoza, Spain
[5]Departamento de Ecología. Universidad Complutense de Madrid. Madrid, Spain
[6] Department of Microbiology, Division of Parasitology. New York University School of Medicine, New York, NY, USA

*e-mail: Corresponding tifar@ucm.es

A. Model 1 Equations SM.1 below describe the population dynamics of T cells and pathogens during an acute infection:

$$\begin{cases} T''(t) = -kT(t) + \lambda P(t) \\ P'(t) = \alpha P(t) - \beta T(t)P(t) \\ T(0) = 0 \\ T'(0) = 0 \\ P(0) = P_0 \geq P_m \end{cases} \quad \text{, for } T \geq 0, P \geq P_m \tag{SM.1}$$

where $T(t)$ and $P(t)$ are, respectively, the number of effector T cells and pathogens at time t and k, λ, α, β, P_0 and P_m are positive parameters. Parameter k is the elastic constant of the T cell population and represents the inclination of the population to recover its initial equilibrium state. Parameter λ is related to the affinity of the TCR for its cognate antigen. The higher the affinity, the higher the force exerted by the pathogen on the T cell population. Parameters α and β are the growth rate and the removal rate of the pathogen population respectively. Parameter P_m represents the minimum population size for which the pathogen is viable. Figure 5 below represents the behavior of model SM.1. We assume that effector T cells do no exist before the infection (initial condition $T(0) = 0$) and an initial dose of pathogens $P(0) = P_0$. Equations 2 are valid for positive values of $T(t)$ and while the pathogen population remains above the threshold value P_m. If the first condition is violated, the pathogen is tolerated by the immune response and the simulation ends. On the other hand, if the pathogen falls below P_m the infection has been controlled and the population of effector T cells is restored to its initial value by the intrinsic elastic force. In this case, the simulation ends when the population of T cells drops back to zero.

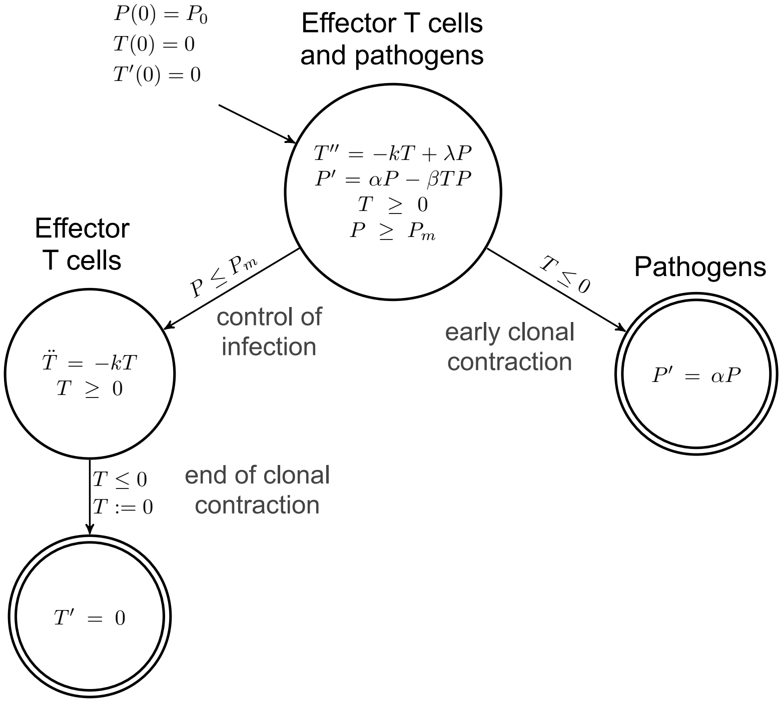

Figure SM.1: Schematic representation of the behavior of model. Equations SM.1 describe the situation after T cell activation. The population of effector T cells is initially zero and expands driven by the antigenic force. If effector T cells disappear before the end of the infection (early clonal contraction), the pathogen escapes the action of the immune response. On the other hand, if the pathogen falls below P_m the infection has been controlled. In this case the pathogen population and hence the antigenic force disappear, and the population of effector T cells is restored to its initial value by the intrinsic elastic force. In this case the simulation continues until the end of clonal contraction.

B. Simultaneous response of several clones Equations SM.1 can be extended to consider the simultaneous activation of several clones of T cells:

Equations SM.2 allow to model differences in TCR/epitope affinities by assigning different values to parameters λ_i. They also considerer potential different elastic responses in activated clones (parameters k_i). As with equations SM.1, the pathogen is controlled if its population drops below the value P_m. On the other hand, the pathogen escapes the immune response if all clones T_i disappear.

$$\begin{cases} T''_i(t) = -k_i T_i(t) + F_i(t), \text{ for } i = 1, \ldots, n \\ P'(t) = \alpha P(t) - \sum_{i=1}^{n} \beta_i T_i(t) P(t) \\ P(0) = P_0 \\ T_i(0) = T_{i0} > 0, \text{ for } i = 1, \ldots, n \\ T'_i(0) = 0, \text{ for } i = 1, \ldots, n \end{cases} \tag{SM.2}$$

for $P \geq P_m$, $\sum_{i=1}^{n} T_i(t) \geq 0$, where $T_i(t)$ is the population of the i-th clone at time t. The antigenic force perceived by each clone of T cells is assumed to be proportional to the relative population of the clone:

$F_i(t) = \lambda_i \frac{T_i(t)}{\sum_i T_i(t)} P(t)$

C. Numerical analysis of equations SM.1 In order to allow for an exhaustive analysis of equations SM.1, we will express them in the following non-dimensional form:

$$\begin{cases} T''(t) = -x(t) + y(t), \\ P'(t) = \alpha^* P(t) - \beta^* T(t) P(t) \quad \text{for } T \geq 0, P \geq P_m^*, \\ P(0) = 1, T(0) = 0, T(0) = 0 \end{cases} \tag{SM.3}$$

where $T(t)$ and $P(t)$ are the number of effector T cells and pathogens at time t and:

$$\alpha^* = \frac{\alpha}{\sqrt{k}}, \beta^* = \frac{\beta\lambda P_0}{k\sqrt{k}} \text{ and } P_m^* = \frac{P_m}{P_0}.$$

For fixed values of the elastic parameter k and the initial dose of pathogens P_0, parameter α^* is proportional to the pathogen growth rate and parameter β is proportional to both the clearance rate and the affinity of the TCR for its cognate epitope. The code for numerical simulations of equations SM.3 was written in Mathematica 7 (Wolfram Research) and is available in the accompanying file **code.nb** (see figure SM.2 and Listing 1 below).

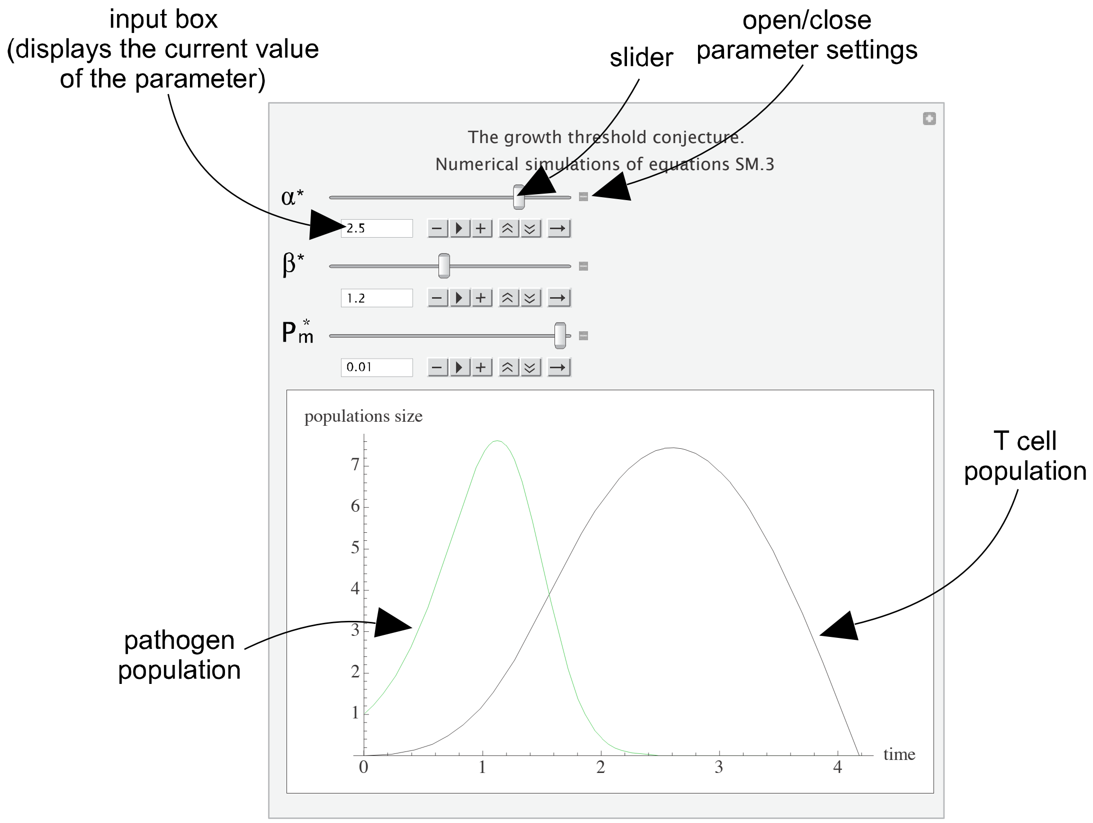

Figure SM.2: Interactive graphic output of the accompanying Mathematica source code. The value of the parameters α^*, β^* and P_m^* can be set by means of sliders or typed in the corresponding input boxes. The pathogen population is displayed in red if pathogens scape the action of T cells and in green otherwise, and T cell population is displayed in black.

Listing 1: Mathematica code for numerical integration of equations SM.3

```
ClearAll[simulation,T,V,P,t,\[Alpha],\[Beta]];
simulation[{\[Alpha]_,\[Beta]_}, {T0_,V0_,P0_},Pm_,t0_, tfin_ ]:=
  (output = {}; pathogenControled = 0;
   Reap[
    NDSolve[{
      T'[t] == V[t],
      V'[t] == - T[t] + P[t] ,
      P'[t] == \[Alpha] P[t] - \[Beta] T[t] P[t],
      T[t0] == T0,V[t0] == V0,P[t0] == P0}, {T,P,V}, {t,t0, tfin },
      EvaluationMonitor :>
      (Clear[t1,T1,V1,P1];
       {t1,T1,V1,P1} = Sow[{t,T[t],V[t], P[t ]}];
       AppendTo[output, {t1,T1,V1,P1}];
       If [T1 < 0 ,Goto[end]];
       If [P1 < Pm,
        pathogenControled = 1;
        ClearAll[t,T,V,P];
        Reap[
         NDSolve[{
           T'[t] == V[t],
           V'[t] == - T[t],
           T[t1] == T1,
           V[t1] == V1}, {T,V}, {t, t1, tfin },
          EvaluationMonitor :>
          (Clear[t2,T2,V2];
           {t2,T2,V2} = Sow[{t,T[t],V[t ]}];
           AppendTo[output, {t2,T2,V2,0}];
           If [T2 < 0,
            Goto[end]];) ]]];
      )
     ]
    ];
   Label[end];
   displayOutput = Union[output, SameTest -> (First[#1] == First[#2] &)];
   color[1] = Green; color[0] = Red;
   Show[{ListLinePlot[Map[{#[[1]], #[[2]]} &, displayOutput],
      PlotStyle -> Black, PlotRange -> {0, Automatic}],
     ListLinePlot [Map[{#[[1]], #[[4]]} &, displayOutput],
      PlotStyle -> color[pathogenControled], PlotRange -> {0, Automatic}]},
    PlotRange -> {0, Automatic},
    AxesLabel -> {"time", "populations␣size"},
    LabelStyle -> Directive["Times", 15], ImageSize -> 500]
   );
Manipulate[simulation[{\[Alpha], \[Beta]}, {0, 0, 1}, Pm, 0,
  20], {{\[Alpha], 2.5}, .1, 3}, {{\[Beta], 1, .1, 3}, {{Pm, .001, .00001, .01},
 FrameLabel -> {"", "", "The␣growth␣threshold␣conjecture.\nNumerical
␣simulations␣of␣equations␣SM.3"}, LabelStyle -> Directive["Times", 14]]
```
